# Immunocytochemical Evidence of the Localization of the Crumbs Homologue 3 Protein (CRB3) in the Developing and Mature Mouse Retina

**DOI:** 10.1371/journal.pone.0050511

**Published:** 2012-11-30

**Authors:** Saúl Herranz-Martín, David Jimeno, Antonio E. Paniagua, Almudena Velasco, Juan M. Lara, José Aijón, Concepción Lillo

**Affiliations:** 1 Institute of Neurosciences of Castilla y León (INCyL), Institute of Biomedical Research of Salamanca (IBSAL), Cell Biology and Pathology, University of Salamanca, Salamanca, Spain; 2 Centro de Investigación del Cáncer-IBMCC (CSIC-USAL), University of Salamanca, Campus Unamuno, Salamanca, Spain; University of Houston, United States of America

## Abstract

CRB3 (Crumbs homologue 3), a member of the CRB protein family (homologous to the *Drosophila* Crumbs), is expressed in different epithelium-derived cell types in mammals, where it seems to be involved in regulating the establishment and stability of tight junctions and in ciliogenesis. This protein has been also detected in the retina, but little is known about its localization and function in this tissue. Our goal here was to perform an in-depth study of the presence of CRB3 protein in the mouse retina and to analyze its expression during photoreceptor ciliogenesis and the establishment of the plexiform retinal layers. Double immunofluorescence experiments for CRB3 and well-known markers for the different retinal cell types were performed to study the localization of the CRB3 protein. According to our results, CRB3 is present from postnatal day 0 (P0) until adulthood in the mouse retina. It is localized in the inner segments (IS) of photoreceptor cells, especially concentrated in the area where the connecting cilium is located, in their synaptic terminals in the outer plexiform layer (OPL), and in sub-populations of amacrine and bipolar cells in the inner plexiform layer (IPL).

## Introduction

Mutations in the *CRB1* gene (Crumbs homologue 1) have been linked to several human retinal dystrophies, including type 12 retinitis pigmentosa (RP12) and Leber congenital amaurosis (LCA) [1], [2]. RP12 is a specific form of retinitis pigmentosa that causes night blindness and loss of visual field in the first ten years of the duration of the disease [1], [3]. LCA is a rare inherited eye disease that appears at birth or early in life, affecting sight and showing other related clinical signs within the first few years of life [4].

Crumbs is a transmembrane protein that was initially identified in *Drosophila*. In this organism, Crumbs plays a crucial role in the establishment and maintenance of cell polarity during the development of several types of epithelial cells [5]. Also, in the *Drosophila* photosensitive organ, the rhabdomere, Crumbs controls the integrity of adherens junctions [6]. To date, three CRB proteins have been identified in mammals: CRB1, CRB2 and CRB3 [7]. In the mouse, CRB1 is only present in the retina and brain [8]; CRB2 mRNA has been found in the retina, RPE/choroid, brain and in other tissues at very low levels [9]; whereas CRB3 is expressed in different epithelium-derived cell types, including the retina [10], [11], [12].

The three CRB proteins share similar short intracellular domains whose role is to organize a highly structured protein scaffold, involving members of the MAGUK family. CRB1 and CRB2 have different and very long extracellular domains, whereas the one in CRB3 is practically non-existent [7]. The localization of the CRB1 protein in the retina of mammals has been extensively studied and is known to be located in the subapical region (SAR) of the outer limiting membrane (OLM) [13], [14], playing an important role in the maintenance of adherens junctions in the OLM, in the polarization of photoreceptor cells, and in the prevention of retinal disorganization after damage due to exposure to excessive light [7], [14], [15] However, the roles of CRB2 and CRB3 in the retina have received little attention, and it remains unknown whether there is any retinal disease related to mutations in the *CRB2* and/or *CRB3* genes, although it does seem that mutations in the CRB2 protein would not be responsible for any of these retinal dystrophies [9]. Additionally, the localization of CRB2 and CRB3 proteins in the retina remains unclear. Regarding CRB2, some studies have demonstrated its mRNA expression in different layers of the retina [9], but the protein has been only localized in the OLM, in both Müller and photoreceptor cells [14], [16].

It has been reported the presence of CRB3 in the OLM as well, in both photoreceptors and in Müller cells [14], [16]. Other authors have suggested that this protein could also be present in the OPL of the retina [17], although little is known about the cells where this protein might be expressed. As mentioned above, CRB3 is also expressed in different epithelium-derived cell types, where some investigators have reported that CRB3 is involved in regulating the establishment and stability of the tight junctions [11], [18], [19], a function that still needs to be investigated in the mammalian retina. Moreover, an alternative CRB3 protein isoform with a sequence ending in CLPI has been described [20]. This isoform seems to play an important role in the ciliogenesis of primary cilium kidney epithelial cells, and the lack of CRB3 protein leads to the absence of cilia in these cells [20], [21]. Photoreceptor cells have a non-motile primary cilium joining the IS with the outer segment (OS), this being necessary for the intracellular protein transport that occurs among both segments. It has been proposed, but not demonstrated, that the CRB3 protein might be localized in the connecting cilium of these cells, where it could also be involved in ciliogenesis [17]. Nevertheless, this possibility remains to be explored.

In the present study we attempted to gain further insight into the localization of CRB3 in the mouse retina by means of double immunofluorescence and Western blot analyses. We re-evaluated its expression in the retina by exploring its presence in this tissue from the stage of P0 until adulthood, since both the OPL and the connecting cilium start to form around these stages of post-natal growth: the connecting cilium develops at P0, being approximately 0.5 µm in length at P1 [22], and the OPL at P4–P5 [23], [24]. Here we show evidences that CRB3 is present all along the inner segment of the photoreceptor cells, where especially concentrates in the connecting cilium area. Also, we found this protein in the OPL and in the IPL, a data that had never been reported before.

## Materials and Methods

All procedures used in this work were in accordance with the guidelines of the European Communities Council Directive (86/609/EEC and 2003/65/EC) and Spanish legislation for the use and care of animals (RD 1201/2005). All the details of the study were approved by the Bioethics Committee of Salamanca University (CBE/30/07/08).

### Animals and Fixation

We used 10 P0 and P4 animals and 30 adult (P90) wild-type mice (C57BL/6J) that were anesthetized with ketamine (100 mg/kg). Adult mice were perfused transcardially with a solution containing 4% paraformaldehyde and 0.2% picric acid in 0.1 M phosphate buffer (PB) at pH 7.4, and post-fixed by immersion for 2 h at room temperature (RT) in the same solution. Then, the eyes were washed in PB and the lens was removed. P0 and P4 mouse eyes were enucleated and fixed overnight (ON) at 4°C by immersion in the same fixative solution, and then washed in PB.

### Western Blot Analyses (WB)

The whole mouse retina was lysed in 1 ml of RIPA buffer (Santa Cruz Biotechnologies®), which was supplemented with a proteases inhibitor cocktail (1∶100, Sigma-Aldrich®). The amount of protein was measured with a Bradford’s assay mixed with 20% Bio-Rad Protein Assay Dye Reagent Concentrate (Bio-Rad laboratories™) and absorbance at 595 nm was measured with a LT4000 Microplate reader (Labtech™). Following this, we boiled 50 µg of proteins dissolved in sample buffer (2% sodium dodecyl sulphate (SDS), 10% glycerol, 700 mM β-mercaptoethanol, 62.5 mM Tris-HCl pH 6.8, 0.05% bromophenol blue), which were later chilled in ice and loaded on a 14% SDS-polyacrylamide gel under reducing conditions. After electrophoresis, proteins were transferred to nitrocellulose membranes, and immunolabeled overnight at 4°C with 1∶500 anti-CRB3 antibodies. After several washes with tris-buffered saline (TBS), the membranes were incubated with 1∶5000 anti-goat IgG conjugated with alkaline phosphatase (Jackson ImmunoResearch™) for 60 min at RT, washed with TBS, and stained with NBT (Nitro-blue-tetrazolium, Roche Applied Science™) and BCIP (5-bromo-4-chloro-3-indolyl-phosphate, Roche Applied Science™). As loading control we used β-actin (1∶5000 dilution). We also carried out a peptide competitive assay as a negative control, where the anti-CRB3 antibody was incubated with 0.2 mg/ml of the antigen fusion protein for 60 min, and this mixture was used instead of the primary antibody dilution.

### Immunofluorescence

The eyeballs were cryoprotected using a graded series of sucrose (10%, 20% and 30%), embedded in Tissue-Tek™ O.C.T., and 14-µm transverse sections were obtained in a cryostat. Autofluorescence was quenched with 0.25 g/l sodium borohydride in 0.1 M phosphate-buffered saline, pH 7.4 (PBS). Sections were then rinsed in PBS with 0.02% Triton Tx-100 (Sigma-Aldrich™) (PBS-Tx) and blocked for 1 h in a solution with 1% bovine serum albumin (BSA) and 5% normal serum in PBS-Tx. Sections were then incubated overnight at 4°C with the primary antibodies and/or peanut agglutinin marker (Table 1) in a solution containing 1% BSA and 2% normal serum in PBS-Tx. Following this, sections were washed with PBS and incubated for 1 h at RT with 1∶250 Cy2 (and Cy3 for the double labeling) fluorescent secondary antibodies (Jackson ImmunoResearch™) in PBS. Sections were mounted using Prolong® Gold antifading reagent (Invitrogen™). Negative controls without primary or secondary antibody were also performed. We also carried out a peptide competition assay by incubating some sections with 0.1 mg/ml of the original antigen fusion protein together with the CRB3 antibody to discriminate any possible background labeling.

**Table 1:** Primary antibodies.

Antigen	Antiserum	Source, catalog number	Working dilution	Labeling
Acetylated tubulin (AT)	Mouse anti-AT	Sigma-Aldrich, T7451	1∶2500	Müller cells
Bassoon	Mouse anti-bassoon	Stressgen, VAM-PS003	1∶5000	Presynaptic terminals
β-catenin	Mouse anti-β-catenin	BD Transduction Laboratories, 610153	1∶300	Adherens junctions
Blue opsin	Rabbit anti-blue opsin	Millipore, AB5407	1∶1000	Blue cones (S cones)
Calbindin (CB)	Rabbit anti-CB	Swant, CB-38a	1∶2000	Horizontal, amacrine and ganglion cells
Calretinin (CR)	Rabbit anti-CR	Swant, 7699/3H	1∶10000	Amacrine and ganglion cells
Cellular retinaldehyde-bindingprotein (CRALBP)	Mouse anti-CRALBP	Abcam, ab15051	1∶400	Müller cells
CRB3	Goat anti-CRB3	Santa Cruz Biotechnologies, sc-29706	1∶500	
Glutamate decarboxylase 65/67(GAD 65/67)	Rabbit anti-GAD65/67	Millipore, AB1511	1∶100	Amacrine cells
Giantin	Rabbit anti-giantin	Abcam, ab24586	1∶1500	Golgi complex
Guanine nucleotide-binding protein G(I)/G(S)/G(T) subunit beta-3 (GNB3)	Rabbit anti-GNB3	Sigma-Aldrich, HPA005645	1∶50	Cone bipolar cells
MAGUK p55 subfamily member 4 (MPP4)	Rabbit anti-MPP4	Designed in our laboratory and producedby Genosphere Biotechnologies	1∶1000	Photoreceptor synaptic terminals and SAR of the OLM
Protein kinase Cα (PKCα)	Rabbit anti-PKCα	Sigma-Aldrich, P4334	1∶5000	Rod bipolar cells
Recoverin	Rabbit anti-recoverin	Millipore, AB5585	1∶2000	Photoreceptor cells
Red/Green opsin	Rabbit anti-red/green opsin	Millipore, AB5405	1∶2000	Red-green cones (L and M cones)
Rhodopsin	Mouse anti-rhodopsin	Abcam, ab54717	1∶1000	Rods
Synaptophysin	Mouse anti-synaptophysin	Sigma-Aldrich, S5768	1∶5000	Synaptic vesicles
Tyrosine hydroxylase (TH)	Rabbit anti-TH	Jacques Roy Institute	1∶5000	Amacrine cells
Vesicular glutamate transporter 1 (VGLUT1)	Rabbit anti-VGLUT1	Synaptic Systems, 135302	1∶500	Bipolar and photoreceptor synaptic terminals

### Antibody Production

The NCBI database (http://www.ncbi.nlm.nih.gov) was used to choose a unique antigenic amino acid sequence of the mouse MAGUK p55 subfamily member 4 protein (MPP4) to design an antibody. The sequence, which included amino acids 586–599, identical to the same region of the MPP4 human protein, was sent to Genosphere Biotechnologies to generate and purify the polyclonal antibodies.

### Imaging

All images were obtained with a laser scanning spectral confocal microscope (Leica TCS SP2) with the pinhole set at 1.0 Airy Units and 40× (numerical aperture:1.25) and 63× (numerical aperture:1.32) immersion oil objectives. The laser lines 488 nm and 543 nm were used to excite Cy2 and Cy3 fluorochromes respectively. Both channels were captured in sequential mode and all images are single optical sections. The brightness and contrast in all original images were further processed and adjusted with Adobe Photoshop CS5 software and Leica Confocal Software.

## Results

**Figure 1:**
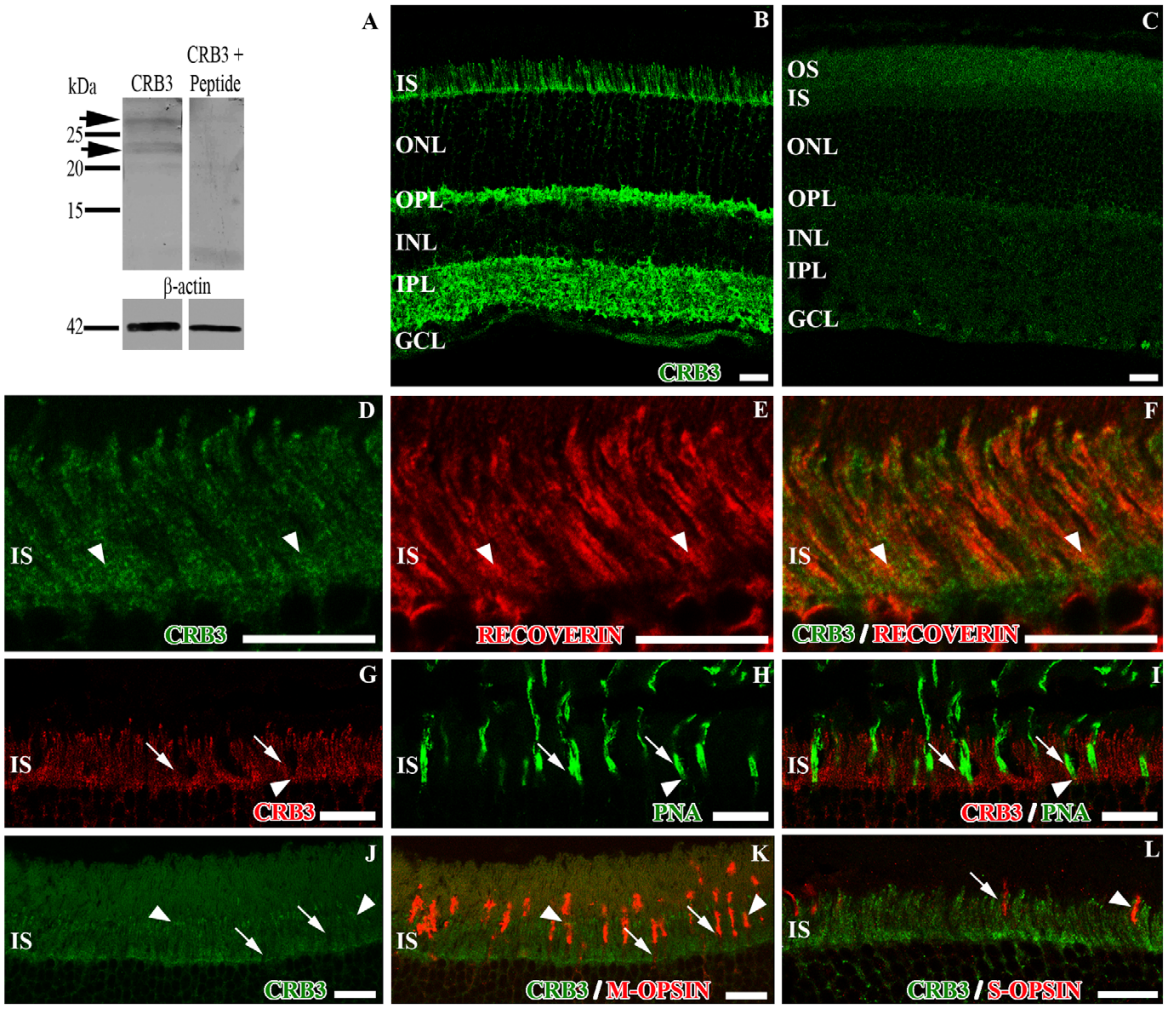
Detection of CRB3 in the mouse adult retina and localization in photoreceptors IS. **A**, western blot analysis showing the expression of CRB3 in retina lysates. The CRB3 protein is detected as two bands (arrows) between 20–28 kDa. In the peptide competitive assay, the specific labeling disappears. β-actin was used as loading control. **B**, CRB3 immunoreactivity in the mouse retina. **C**, peptide competition assay for CRB3 where all the labeling disappears. **D–L**, double immunofluorescence for CRB3 (green in **D**, **F**, **J**, **K** and **L;** red in **G**, **I**) and recoverin (red in **E–F**), PNA (green in **H–I**), M-opsin (red in **K**) or S-opsin (red in **L**). CRB3 partially colocalizes with all of these markers in the IS. The arrowheads in **D–L** point out to some areas of colocalization. The arrows in **G–L** point out to regions where these markers do not colocalize. OS, photoreceptor outer segments; IS, photoreceptor inner segments; ONL, outer nuclear layer; OPL, outer plexiform layer; INL, inner nuclear layer; IPL, inner plexiform layer; GCL, ganglion cell layer. Scale bars: 20 µm.

We employed Western Blot (WB) analysis to test the specificity of the antibody used in the present work for the recognition of the CRB3 protein in the mouse retina. In retina lysates we detected two bands with an approximate molecular weight between 20–28 kDa (Fig. 1A), which are the reported molecular weights for the two different glycosylated protein isoforms; the isoform a, with an ERLI-ending (CRB3-ERLI), and the isoform b, with a CLPI-ending (CRB3-CLPI) [12], [20], [21].

According to the immunofluorescence assays, the staining pattern shows that CRB3 is localized in both synaptic layers of the retina: the OPL and throughout the entire IPL, although the IPL is not labeled homogeneously (Fig. 1B). We also found labeling for CRB3 in the inner photoreceptor segments, where it is especially intense in the region between the inner and outer segments and close to the OLM (Fig. 1B). The specificity of the anti-CRB3 antibody was assessed with a peptide competitive assay (Fig. 1C, Fig. S1A–B).

### CRB3 Expression in Rod and Cone Outer and Inner Segments

To further analyze the localization of CRB3 in photoreceptor cells we used antibodies to detect recoverin (present in all photoreceptor cells), two different antibodies to distinguish M from S cones in mouse and PNA (to label all cones). Recoverin is a cytoplasmic protein located in both types of photoreceptor cells, in their IS, cell bodies in the ONL and in their synaptic terminals in the OPL (Fig. 1E). The double labeling recoverin/CRB3 shows uniform staining of the rod and cone IS for the two markers (Fig. 1D–F). PNA binds to carbohydrates in the external surface of the plasma membrane of cones at the level of their IS, in some of the OS and at the base of the cone pedicles in the OPL (Fig. 1H). The antibody against the red/green opsin labels the M cones in the mouse retina, being present throughout both segments, in the ONL surrounding the nuclei and in some of their synaptic terminals (Fig. 1K). The blue opsin labeling showed that this protein is only present along the S cone outer segments (Fig. 1L). The double labeling for these markers and CRB3 revealed a limited overlap of these proteins in the cone sheaths (arrowheads in Fig. 1G–L), although generally, the labeling for the CRB3 protein showed some gaps at the level of the IS of the photoreceptor cells in which the PNA or opsin staining was distinguishable (arrows in Fig. 1G–L).

**Figure 2:**
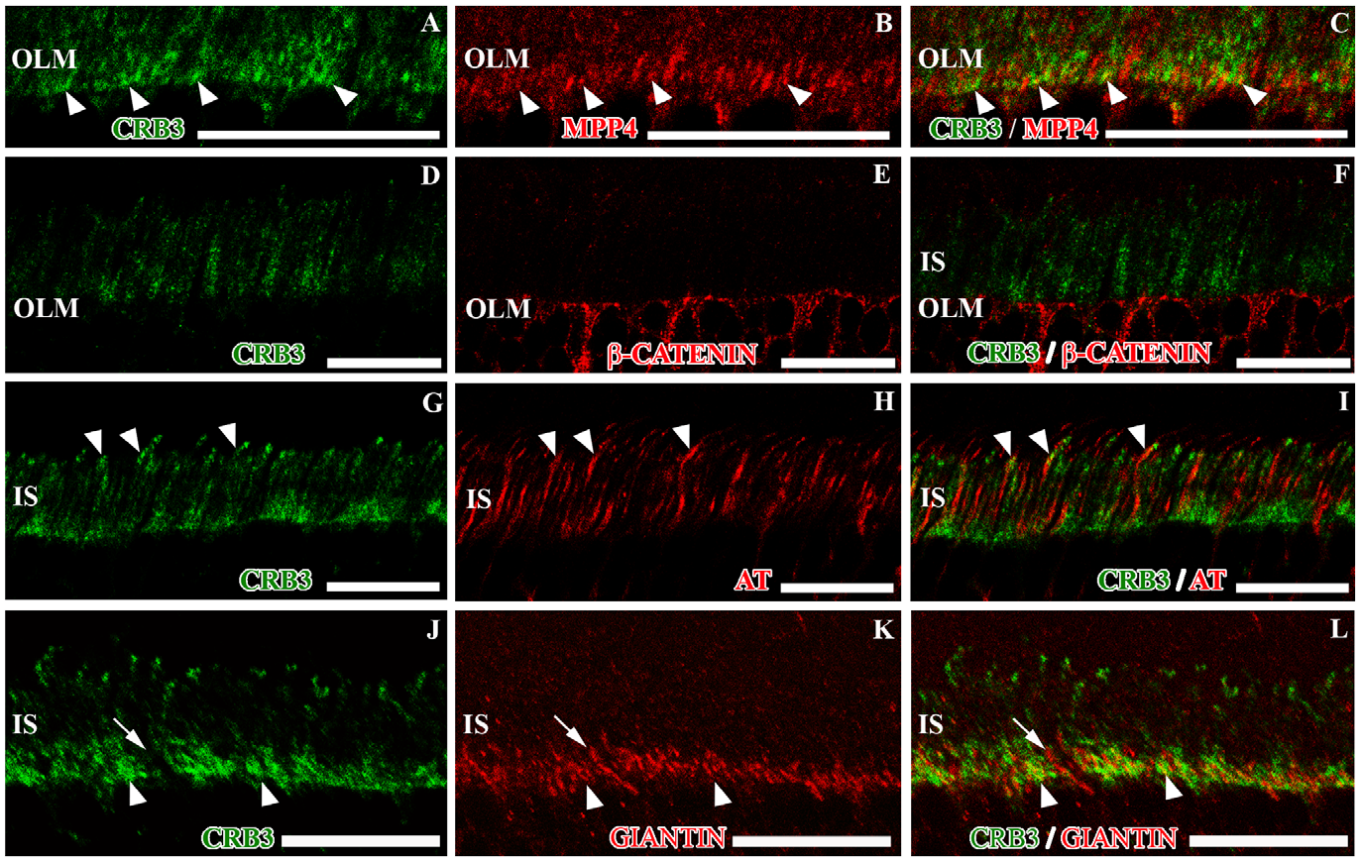
CRB3 in the photoreceptor IS area of the adult mouse. Double immunolabeling for CRB3 (green) and MPP4 (red in **B–C**), β-catenin (red in **E–F**), acetylated tubulin (AT, red in **H–I**) or giantin (red in **K–L**). CRB3 colocalizes with MPP4 in the photoreceptor inner segments (arrowheads in **A–C**); nevertheless CRB3 in not present in the OLM adherens junctions, which are labeled for β-catenin (**D–F**). **G–I**, CRB3 seems to surround most of the AT positive connecting cilia, and in some cases, both proteins colocalize (arrowheads in **G–I**). **J–L**, CRB3 colocalizes with the Golgi complex marker giantin in the photoreceptor cells IS (arrowheads), but there are some giantin positive profiles where CRB3 is not present (arrows). IS, photoreceptor inner segments; OLM, outer limiting membrane. Scale bars: 20 µm.

To further investigate the presence of CRB3 in the area close to the OLM and at the tip of the IS, we used different antibodies against proteins known to be present in these regions, such as MPP4, β-catenin, acetylated tubulin, rhodopsin and giantin, which is expressed in the Golgi apparatus (Fig. 2B, E, H, K; Fig. S1C, E).

MPP4 is a protein present in the OPL and in the SAR of the OLM, where it has been identified as a member of the protein scaffold complex formed by members of the CRB and the MAGUK protein families [25], [26], [27]. In the OLM, our antibody shows a weak labeling for MPP4 (Fig. 2B; Fig. S1C), and the peptide competitive assay demonstrated its specificity (Fig. S1D). Double labeling for CRB3 and MPP4 showed that these two proteins partially colocalized at the level of the OLM (Fig. 2A–C).

The OLM is formed by the adherens junctions established between Müller and the photoreceptor cells, where β-catenin is present (Fig. 2E). The CRB3/β-catenin double labeling revealed that there was no colocalization of these two proteins in this area, since CRB3 seemed to be located in the SAR, apically to β-catenin (Fig. 2D–F).

The outer and inner segments of the photoreceptor cells are associated through a connecting cilium, which is situated at the tip of the IS, where the CRB3 staining seemed to be more intense (Fig. 2G). The acetylated tubulin protein was present in the cytoskeletal structure that formed the cilium in this area (Fig. 2H). The CRB3/AT double immunolabeling showed that both proteins were located in the cilium area, and although there were some points of colocalization, most of the CRB3 labeling seemed to be adjacent the AT-labeled connecting cilia (Fig. 2G–I). Besides, since the rhodopsin molecule is only located in the rods OS, the double labeling CRB3/rhodopsin showed colocalization at the tip of the IS (Fig. S1E–F).

In the region close to the OLM, since CRB3 did not colocalize with β-catenin, we attempted to identify the cell compartment where CRB3 could be present. The giantin labeling revealed that the Golgi complex of the photoreceptor cells is restricted to this area of the IS (Fig. 2K), and the double labeling CRB3/giantin demonstrated that these two proteins entirely colocalized in this area except for some restricted giantin positive profiles that were negative for CRB3 (Fig. 2J–L). Besides, we have shown that CRB3 partially colocalizes in this area with MPP4 (Fig. 2C), which is present in membranous compartments in the Golgi area of the photoreceptors IS.

### CRB3 Protein Distribution in the OPL

**Figure 3:**
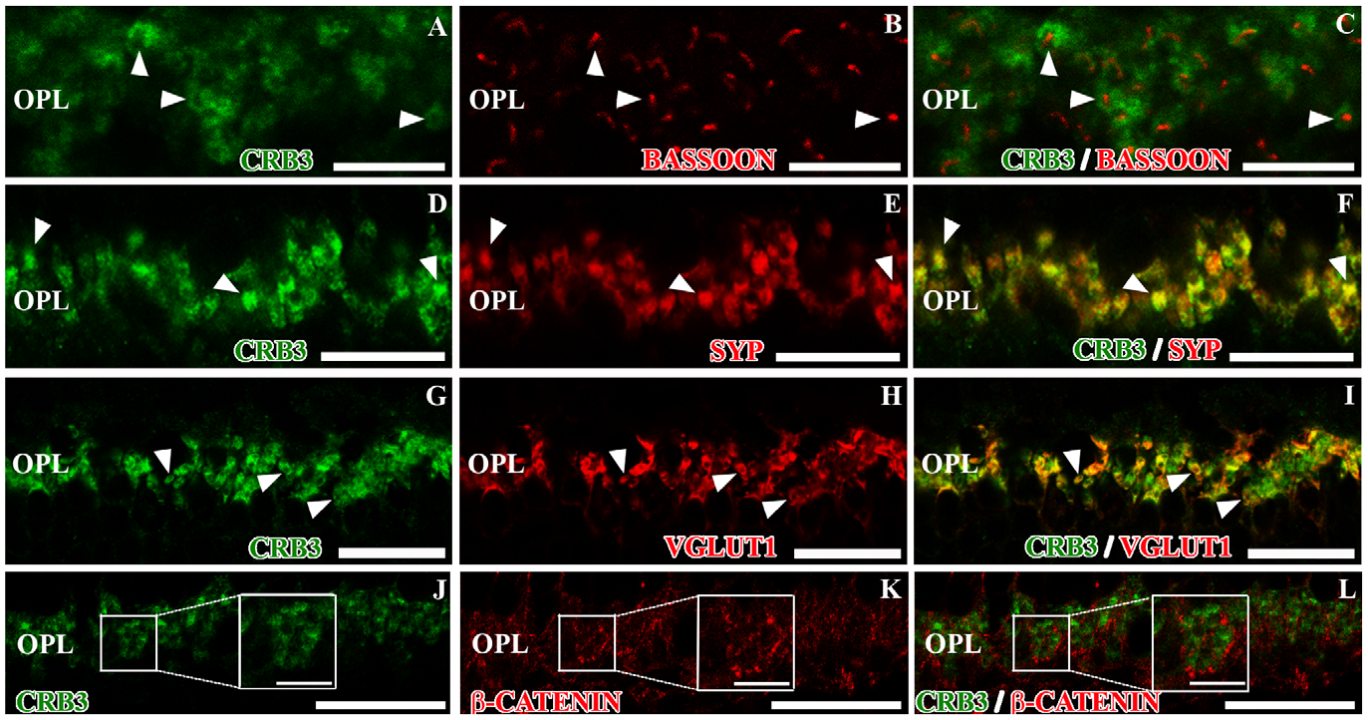
CRB3 positive profiles in the outer plexiform layer of the adult mouse. Double immunofluorescence for CRB3 (green) and bassoon (red in **B–C**), synaptophysin (SYP, red in **E–F**), VGLUT1 (red in **H–I**) and β-catenin (red in **K–L**). **A–F**, CRB3 seems to surround the bassoon labeling (arrowheads in **A–C**) and colocalizes with SYP (arrowheads in **D–F**). **G–I**, VGLUT1 is present in the photoreceptors’ presynaptic terminals (**H–I**), where it colocalizes with CRB3 (arrowheads in **G–I**). **J–L**, β-catenin does not colocalize with CRB3 in the OPL (insets in **J–L**). Insets in J–L: Higher magnifications of the small squares. OPL, outer plexiform layer. Scale bars: 20 µm, 5 µm in inset.

To analyze the localization of CRB3 in the plexiform layers, we performed double labeling experiments for CRB3 and different proteins which are expressed in the synaptic terminals. Bassoon is a protein associated with synaptic ribbons in photoreceptors and bipolar cell terminals and the presynaptic active zone in conventional synapses made by amacrine cells. Synaptophysin is located in the pre-synaptic vesicles. The double labeling CRB3/bassoon demonstrated that in the OPL, CRB3 did not colocalize with bassoon, since it surrounded the bassoon positive photoreceptors synaptic ribbons (Fig. 3A–C). On the other hand, CRB3 and synaptophysin fully colocalized in the synaptic terminals (Fig. 3D–F). VGLUT1 is a vesicular glutamate transporter which in the OPL is located in photoreceptors presynaptic terminals. The CRB3/VGLUT1 double labeling showed that all VGLUT1-positive processes express CRB3, but although both proteins were present in the same terminals, the colocalization was not complete (Fig. 3G–I).

The protein β-catenin is localized in the adherens junctions formed in the synaptic contacts. Since the labeling for CRB3 showed the presence of this protein in the plexiform layers, we analyzed the possible colocalization of these two proteins in the OPL. We found that they did not colocalize, since β-catenin seemed to be located in the gaps left by the CRB3 staining (Fig. 3J–L).

**Figure 4:**
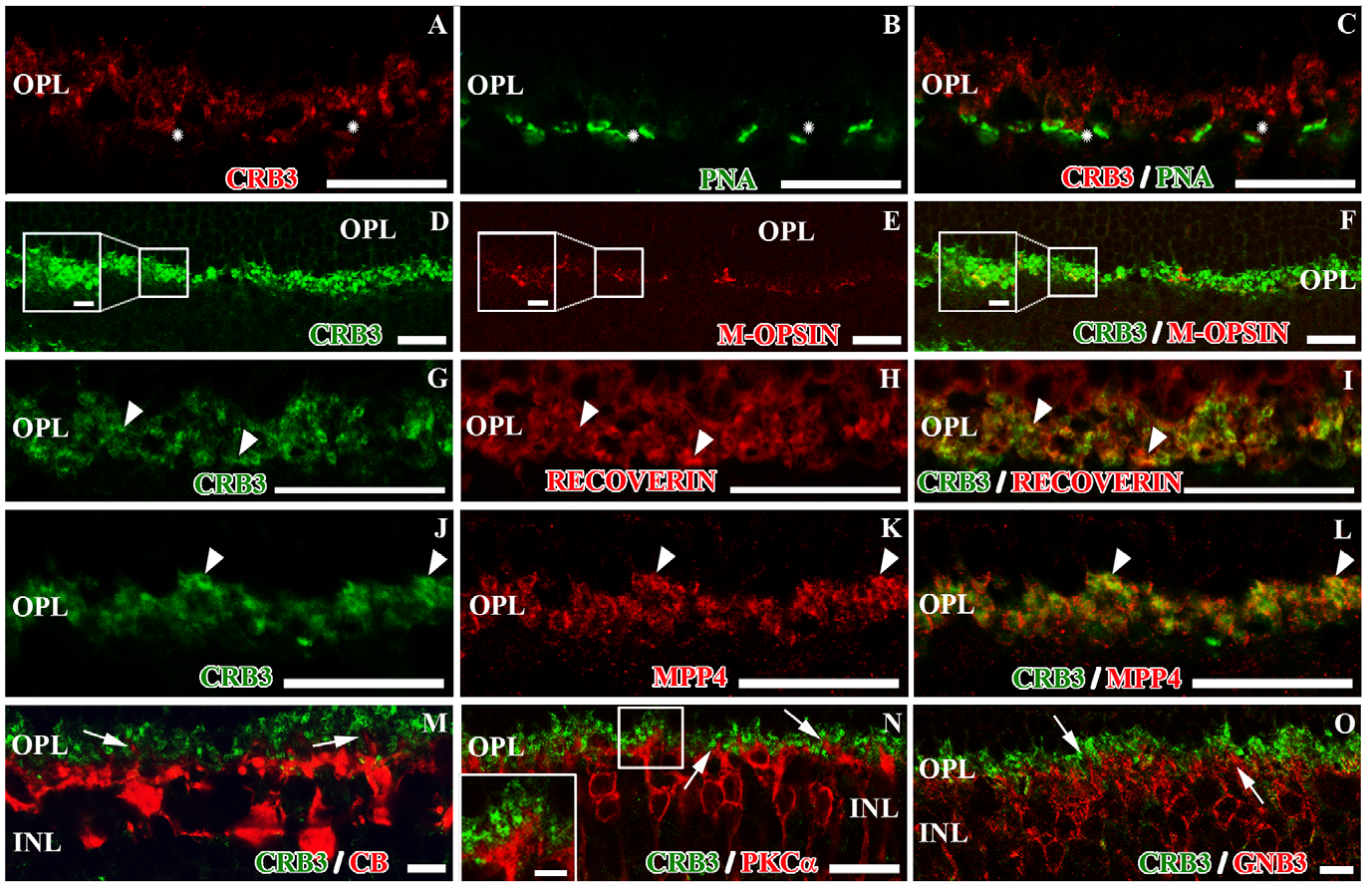
CRB3 positive profiles in the outer plexiform layer of the adult mouse (II). Double immunofluorescence for CRB3 (red in **A**, **C**; green in **D**, **F**, **G**, **I**, **J**, **L**, **M**, **N** and **O**) and markers for different cell types present in the OPL. **A–C**, CRB3 seems to be present at the base of cone terminals (asterisks), positive for PNA (green in **B**, **C**). **D–F**, CRB3 colocalizes with markers for M-opsin (red in **E, F**) (inset in **D–F**). **G–I**, recoverin (red in **H, I**) labels photoreceptors’ presynaptic terminals, in both cones and rods and colocalizes with CRB3 (arrowheads). **J–L**, MPP4 (red in **K, L**) is also localized in both types of photoreceptor presynaptic terminals, where it colocalizes with CRB3 (arrowheads). **M**, CRB3 does not colocalize with CB (red) positive horizontal cells in the OPL (arrows in M). **N–O**, anti-PKCα labels the rod bipolar cells (red in **N**), and anti-GNB3 stains a subset of cone bipolar cells (red in **O**). In the OPL, none of the bipolar cells dendrites contain CRB3. The inset in **N** and the arrows in **N–O** show bipolar cell processes outlined by the CRB3 staining. Insets in D–F and N: Higher magnifications of the small squares. OPL, outer plexiform layer; INL, inner nuclear layer. Scale bars: 20 µm, 2 µm in inset.

Photoreceptor cells establish synaptic contacts with the dendrites of the bipolar and horizontal cells in the OPL. To identify the profiles that express CRB3, we used markers to distinguish the photoreceptor pre-synaptic terminals (PNA, different opsins, recoverin and MPP4) from the dendrites of the horizontal cells (calbindin or CB) or those of the bipolar cells (PKCα and GNB3). At the level of the OPL, some cone specific markers such as PNA or M-opsin stain the base of the cone pedicles. The double labeling with CRB3 and these markers appears to show that CRB3 may be present along the base of the cone terminals (Fig. 4A–F). As mentioned earlier, recoverin is present in the presynaptic terminals of all photoreceptor cells in the OPL, where the double immunofluorescence labeling showed a complete colocalization of these two proteins (Fig. 4G–I). The CRB3/MPP4 double immunolabeling also showed colocalization of these two proteins in the OPL, although some single-labeled profiles were found (Fig. 4J–L). Since recoverin, MPP4 and VGLUT1 are known to be present in rods and cones [27], [28], [29], [30], [31], [32], these observations support the idea that CRB3 might be present in the pre-synaptic terminals of both, cone and rod photoreceptor cells.

**Figure 5:**
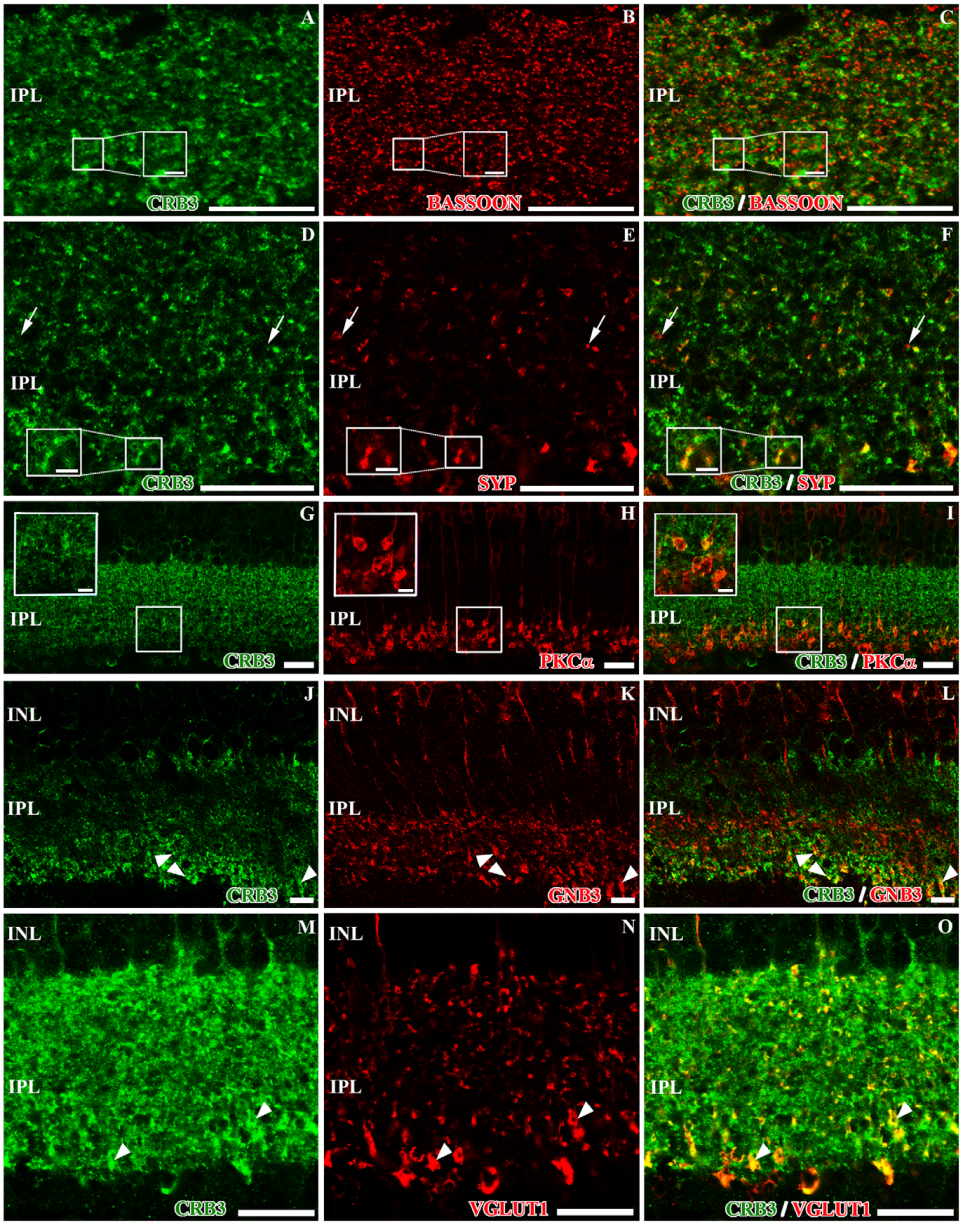
Localization of CRB3 in the inner plexiform layer of the adult mouse. Double immunofluorescence for CRB3 (green) and bassoon (red in **B–C**), synaptophysin (SYP, red in **E–F**), PKCα (red in **H–I**), GNB3 (red in **K–L**) and VGLUT1 (red in **N–O**). **A–F**, CRB3 in the IPL surrounds the bassoon labeling (inset in **A–C**) and colocalizes with synaptophysin (inset in **E–F**), although some single labeled SYP positive profiles are found (arrows in D–F). **G–L**, in the IPL, PKCα is present in the axons of the rod bipolar cells (**H–I**), and GNB3 is present in the axons of a sub-population of cone bipolar cells (**K–L**). CRB3 colocalizes with both proteins in the innermost part of the IPL (insets in **G–I** and arrowheads in **J–L**). **M–O**, VGLUT1 is also present in the bipolar cells’ presynaptic terminals (**N–O**). All VGLUT1 positive processes colocalize with CRB3 (arrowheads in **M–O**), but not the opposite. Insets in A–I: Higher magnifications of the small squares. INL, inner nuclear layer; IPL, inner plexiform layer; GCL. Scale bars: 20 µm, 5 µm in inset (D–F) and 2 µm in inset (G–I).

We explored the possibility that CRB3 could be also present in the dendrites of bipolar and horizontal cells in the OPL. The double immunolabeling CRB3/CB showed no colocalization of the two proteins in the horizontal cells, since the CB staining, although very close to the CRB3 positive profiles, seems to surround them (Fig. 4M). Anti-PKCα is a well-known antibody for rod bipolar cells, and Guanine nucleotide-binding protein 3 (GNB3) is present in the cone-ON bipolar cells. PKCα and GNB3 are distributed throughout the cell body of these cells, extending from the OPL to the IPL (Figs. 4N, O; 5H, K). Double immunolabeling with CRB3 revealed that this protein is not present in the dendrites of the bipolar cells at the level of the OPL (Fig. 4N, O).

### CRB3 Protein Distribution in the IPL

CRB3 immunostaining pattern showed that this protein is also present in the IPL. In this retinal layer, bipolar cells establish synaptic contacts with different subpopulations of amacrine and ganglion cells. Double immunofluorescence experiments for CRB3 and the presynaptic markers used to label the OPL as well, such as bassoon and synaptophysin, showed that CRB3 outlined all the profiles labeled for bassoon (Fig. 5A–C) and colocalized with most of the synaptophysin positive profiles, although some single-labeled elements were found (Fig. 5D–F).

In order to identify whether the different subpopulations of amacrine, ganglion and bipolar cells present in this layer contained the CRB3 protein, we performed double immunolabeling for CRB3 and antibodies against proteins known to be present in these cells, such as PKCα, GNB3, VGLUT1, Glutamate decarboxylase 65 and 67 (GAD 65/67), CB, calretinin (CR) and tyrosine hydroxylase (TH).

All the rod bipolar cell synaptic terminals, as well as their processes in the IPL, were positive for PKCα (Fig. 5H). These synaptic terminals are distributed in the innermost layer of the IPL and, at this level, the PKCα positive terminals colocalized with CRB3 (Fig. 5G–I), where in some profiles, the CRB3 labeling pattern is present as a ring associated with the rod bipolar cell terminals. Likewise, the GNB3 protein was distributed through the entire cell body of the cone-ON bipolar cells, and the double labeling CRB3/GNB3 showed that these two proteins only colocalized in the innermost area of the IPL (Fig. 5J–L). Furthermore, in the IPL, VGLUT1 is located in the bipolar cells synaptic terminals (Fig. 5N), and the double labeling CRB3/VGLUT1 showed that in this layer, all VGLUT1-stained processes were CRB3-positive, but not the opposite (Fig. 5M–O).

**Figure 6:**
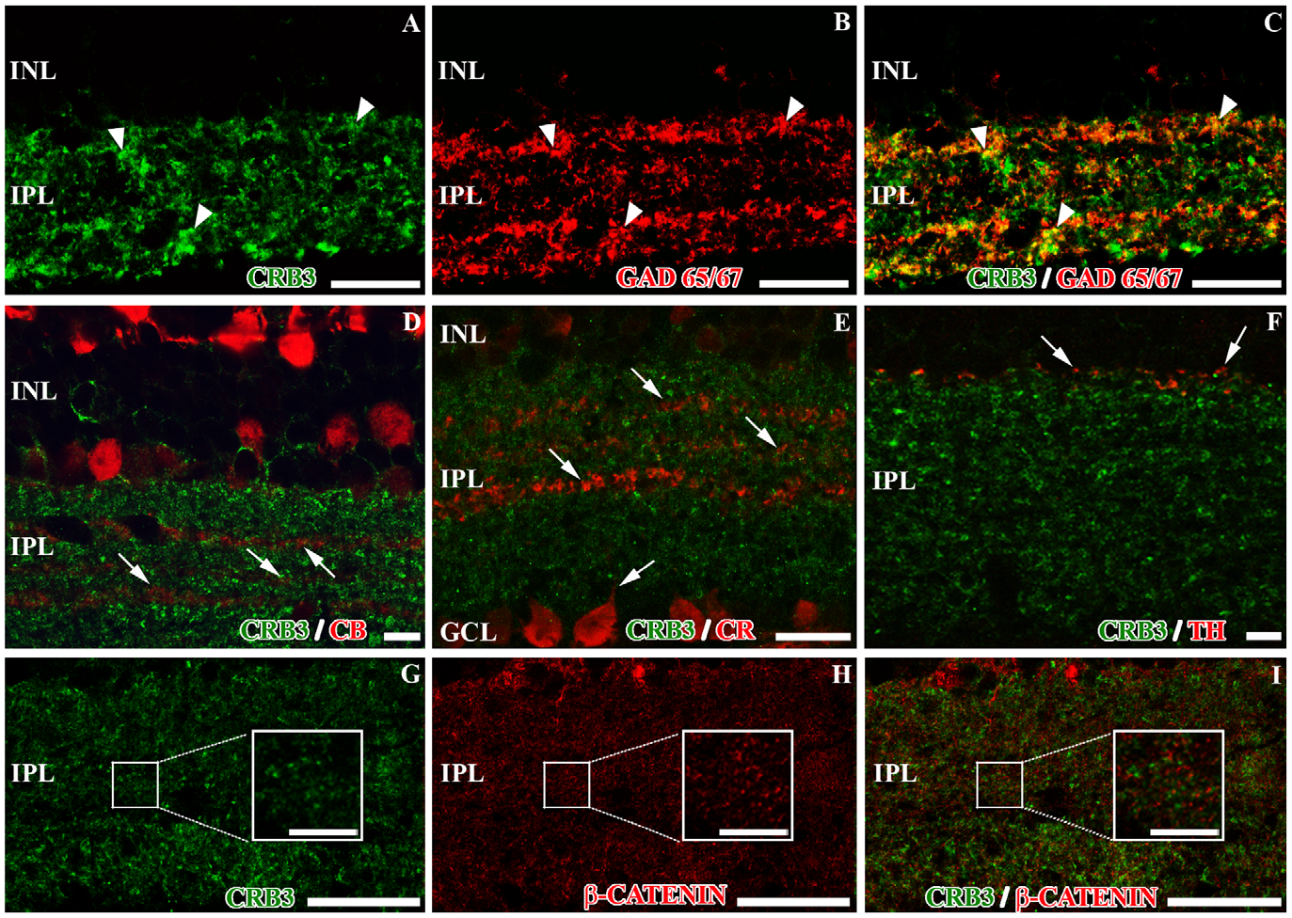
Presence of CRB3 in the inner plexiform layer of the adult mouse (II). Double immunofluorescence for CRB3 (green) and markers for different IPL cell types (red). **A–C**, GAD 65/67 is present in GABAergic amacrine cells (**B–C**). There is a partial colocalization of GAD 65/67 positive profiles and CRB3 (arrowheads). **D–F**, CRB3 does not colocalize with amacrine CB positive profiles in the IPL (arrows in **D**), with the amacrine or ganglion cells CR positive profiles (arrows in **E**) or with the amacrine TH positive processes (arrows in **F**). **G–I**, CRB3 does not colocalize with β-catenin in the IPL (insets in **G–I**). Insets in G–I: Higher magnifications of the small squares. INL, inner nuclear layer; IPL, inner plexiform layer; GCL, ganglion cell layer. Scale bars: 20 µm.

GABAergic amacrine cells account for 25–55% of all amacrine cells, depending on the species [33]. These cells were labeled with antibodies against the GABA-synthesizing enzyme glutamic acid decarboxylase GAD 65/67 (Fig. 6B). The double labeling CRB3/GAD 65/67 showed a high degree of colocalization of the two proteins, although we found some GAD positive processes that did not show CRB3 labeling, and vice versa (Fig. 6A–C).

The calcium-binding proteins CB and CR are present in a subpopulation of amacrine cells located in three strata in the IPL. Besides, CR is a specific marker for starburst amacrine cells [34]. CRB3 seems to be absent from all these populations of cells (Fig. 6D, E). In fact, the CB and CR proteins were present in the gaps left by the CRB3 staining (Fig. 6D, E). CB (not shown) and CR were also present in a subpopulation of ganglion cells, in their cell bodies, and in their processes; where CRB3 was not present (Fig. 6E). TH is present in the large dopaminergic amacrine cells, whose TH-positive processes are distributed in the outermost layer of the IPL (Fig. 6F). The TH/CRB3 double labeling did not reveal colocalization of these two proteins except for some discrete profiles (Fig. 6F).

As in the OPL, β-catenin is present in the adherens junctions established in the synaptic contacts of the IPL. In this layer, and similarly to what we found in the OPL, β-catenin and CRB3 do not colocalize (Fig. 6G–I).

### CRB3 is Present in the IPL and in the Outer Retina at P0 and P4

To analyze CRB3 protein expression during the development of the elements where we detect this protein, we performed double immunolabeling for CRB3 and several proteins known to be present in the mouse retina at P0, when the photoreceptor connecting cilium starts to develop, and at P4, prior to the complete establishment of the OPL.

**Figure 7:**
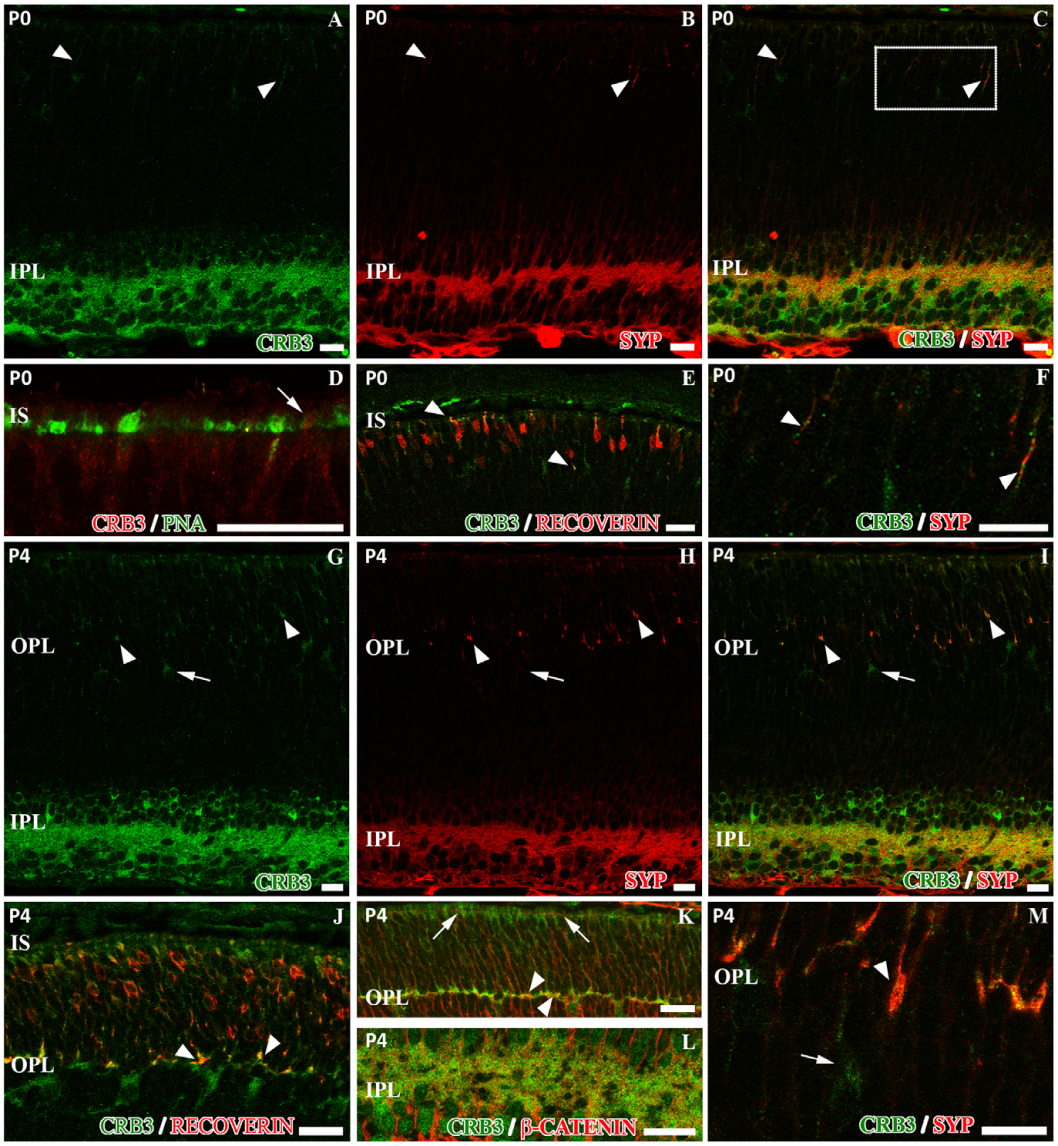
Characterization of the CRB3 protein expression in the mouse retina at P0 and P4 stages. **A**, at P0, CRB3 (green) is present in the outer retina as well as in the IPL. Double immunolabeling for CRB3 and synaptophysin (SYP, red in **B–C** and **F**) showed that CRB3 partially colocalizes with this protein in the IPL (**A–C**), and is also present in the incipient outer retina (arrowheads in **A–C** and inset of **C** in **F**). **D–E**, in the outer retina, PNA (green in **D**) and recoverin (red in **E**) are already being expressed by the developing photoreceptor cells. The colocalization of PNA and CRB3 (red in **D**) is not clear (arrow in **D**); however, recoverin and CRB3 (green in **E**) partially colocalize (arrowheads in **E**). **G**, at P4, CRB3 (green) is present in the IPL, in the developing OPL and in photoreceptors. The double immunolabeling for CRB3 (green in **G**, **I** and **M**) and synaptophysin (SYP, red in **H**, **I** and **M**) shows that in the IPL, CRB3 colocalizes with synaptophysin (**I**). Also, in the incipient OPL, all synaptophysin positive profiles are CRB3 positive (arrowheads in **G–I** and **M**) but others are CRB3 positive and synaptophysin negative (arrows in **G–I** and **M**). **J**, in the developing photoreceptor cells, recoverin (red) is present all along the cell bodies. CRB3 (green in **J**) colocalizes with recoverin in the area where the inner segments are forming and in the developing OPL (arrowheads in **J**). **K–L**, β-catenin (red) does not colocalize with CRB3 (green) at the level of the OLM during development (arrows in **K**). Both proteins partially colocalize in the OPL (arrowheads in **K**) and in the IPL (**L**). IS: photoreceptor inner segments; OPL, outer plexiform layer; IPL, inner plexiform layer. Scale bars: 20 µm.

At P0, some layers of the retina are starting to develop, such as the GCL or the IPL. At this age, synaptophysin was extensively found in the IPL and, with less intensity, in the outer retina (Fig. 7B). CRB3 was also present at P0 at the level of the IPL, and some scattered labeling was also detected in the outer retina (Fig. 7A). In the IPL, synaptophysin and CRB3 colocalized, although all processes positive for synaptophysin were labeled with CRB3, but not the opposite (Fig. 7C). Additionally, in the outer retina, a partial colocalization of these two proteins was observed (Fig. 7C, F). PNA labeled the cones IS during their development at this age (Fig. 7D), and the PNA/CRB3 double labeling revealed that presence of CRB3 in these IS was not definite (Fig. 7D). At P0, recoverin was present in the developing photoreceptor cells (Fig. 7E), as well as in some ganglion cells, as seen in the adult retina (not shown). Double immunolabeling with CRB3 showed a partial colocalization with recoverin in the area where the photoreceptor IS are developing and in some of the photoreceptor cell bodies (Fig. 7E). At P4, the double immunolabeling showed that CRB3 maintained its colocalization with synaptic proteins at the level of the IPL, such as synaptophysin (Fig. 7G–I). At this age, the synaptophysin labeling in the outer retina was stronger than at P0, and there was a partial colocalization with CRB3, since all synaptophysin processes were CRB3-positive, but not the opposite (Fig. 7G–I, M). At P4, recoverin is present throughout the developing photoreceptor cells and in the area where the OPL starts to form (Fig. 7J). As mentioned earlier, CRB3 was present in the area where the photoreceptor IS were growing and in the developing OPL (Fig. 7J). In the incipient OPL, all the processes positive for recoverin were also labeled for CRB3, but not the opposite (Fig. 7J). Moreover, CRB3 was also located in some cells just below the OPL (Fig. 7G, I, J, M). The adherens junctions start to form early during development, so the β-catenin protein is present throughout the retina at these stages. At P4, and unlike in adult mice, we found a partial colocalization of β-catenin and CRB3 in the developing OPL (Fig. 7K), and also in the IPL (Fig. 7L). At this stage, we also observed the distribution of the CRB3 protein in the OLM, where, as in the adult, CRB3 and β-catenin did not colocalize (Figs. 7K; 2F).

## Discussion

### CRB3 Protein Distribution in the Photoreceptors Inner Segments

Using the antibodies described in two publications [11], [12], the few authors who have addressed the distribution of CRB3 in the mouse retina have only located the protein at the level of the OLM, in photoreceptors, and in Müller cells [14], [16] In the present study, we detected CRB3 in Müller cells after performing double immunolabeling with anti-CRALBP, a Müller cell marker (Fig. S2), although we have observed that the majority of the localization is in photoreceptor cells.

Regarding the localization of CRB3 at the tip of the IS, we obtained a punctate staining in the budding IS at P0 and at P4, overlapping with the development of the photoreceptor connecting cilium, which is approximately 0.5 µm in length at P1 [22]. Furthermore, in adult mice we detected CRB3 in the connecting cilium area, where this protein surrounds the staining obtained for the acetylated tubulin protein, which is present in the cilium [35]. To date, few studies have analyzed the role of CRB3 in the ciliogenesis of some epithelial cells in mammals, showing that this protein is required for ciliogenesis [20], [21]. In the work published by Fan et al. in 2007, MDCK cells transfected with shRNA specifically directed towards the CRB3-CLPI isoform did not form the cilia, demonstrating that the CRB3-CLPI isoform is the one implicated in ciliogenesis [20]. However, although the presence of CRB3 in the photoreceptor connecting cilium and the involvement of this protein in its development had been suggested [17], the present work is the first one to demonstrate this possibility. Thus, our results show for the first time the presence of CRB3 protein in the connecting cilium area early from the development of this element.

In the photoreceptor cells there is an important Golgi-based sorting and vesicle trafficking of cilia elements, since the biosynthetic machinery is absent from cilia, so all their molecular components must be synthesized in the organelles located in the IS and transported to the cilium. Recently, it has been demonstrated that many of the components necessary for the correct formation or functioning of the photoreceptor cilium are also located in the Golgi complex, such as some of the intraflagellar transport proteins [36], and the retinitis pigmentosa protein RP2 [37] among others. It has been also reported that some proteins from the scaffolding complex organized by the CRB proteins, such as MPP4, are detected in membranous compartments in the Golgi area of the photoreceptors IS [26]. Additionally, the CRB3-CLPI that has been identified to be located in the cilia membrane in epithelial cells in culture has been also detected in the Golgi complex of these cells during interphase [20]. Therefore, it is not surprising that we detected CRB3 not only in the connecting cilium of the photoreceptor cells, since we seem to detect the cilia-related CRB3-CLPI isoform in the mouse retina, but also in the Golgi complex, where it colocalized with giantin and MPP4, supporting the idea that CRB3 is another component of the cilium that needs to be vesicle-transported to this compartment. The data obtained from the double labeling to distinguish the IS from the two types of photoreceptor cells (opsins and PNA for cones, rhodopsin for rods and recoverin and giantin for both) and CRB3 show that this protein is clearly expressed in the IS of rods, and although we found some colocalization of cone markers and CRB3, the presence in the inner segments of cones is not conclusive. Nevertheless, the staining pattern obtained for MPP4 strongly support the idea that CRB3 may be present in both cone and rod IS.

Since previous studies have shown that CRB3 has a determinant role in the development of the primary cilia in several epithelial tissues [20], [21], and after our own findings, we believe that CRB3 might play an active role during the development of the photoreceptors’ connecting cilium and in IS growth.

### CRB3 Protein in the Adherens Junctions

The Crumbs complex, which includes CRB3, has been described to be associated with the adherens junctions that organize the OLM in the retina [7]. Different studies have shown that one of its members, CRB1, and β-catenin, which is present in these junctions [38], do not colocalize in the OLM, since CRB1 is located in the SAR apically to β-catenin. In the present study, we show that CRB3 did not colocalize with β-catenin either. Furthermore, the initial step in synapse development is the formation of an adherens-like junction [39], [40], where it is known that CRB1 is not present [26], but at P4, CRB3 did show a partial colocalization with β-catenin in the OPL and in the IPL. Also, at P0, when the IPL has started to develop, CRB3 and β-catenin partially colocalized in these layers. Considering this, and bearing in mind the importance of CRB3 in the regulation of cell-cell junctions in several cell types [7], it seems feasible that CRB3 might collaborate with β-catenin in the establishment of adherens junctions in the retinal plexiform layers. Nevertheless, CRB3 might not be necessary for the maintenance of these junctions when they are already established.

### CRB3 in the Plexiform Layers

In the present work we show that the bulk of the CRB3 signal is present in both plexiform layers, a hitherto unreported observation [14], [16]. Previous studies using a Pan-CRB antibody that recognizes all three CRB proteins have suggested the presence of CRB3 and/or CRB2 in the OPL, since CRB1 was undetected in this layer [17], [26]. It is possible that the presence of CRB3 in the plexiform layers had not been reported before due to the use of antibodies raised against different regions of the CRB3 sequence, which probably did not recognize all isoforms. Our WB experiments showed that the antibody used in the present work effectively recognized the two known glycosylated isoforms of CRB3 in the mouse retina [10], [11], [12], [20], [21]. The labeling for CRB3 in the OPL showed that this protein outlines the staining obtained for bassoon, which is localized in the synaptic ribbons in cones and rods in this layer, as well as in bipolar cells and in the active zone of the conventional synapses of amacrine cells in the IPL [41]. Synaptophysin is a protein localized in the membrane of the synaptic vesicles of photoreceptor cells in the OPL, as well as in bipolar and amacrine cell presynaptic terminals in the IPL, in both ribbon and conventional synapses [42]. According to this, we probably detected CRB3 together with synaptophysin in the synaptic vesicles, although since we found some synaptophysin single-labeled profiles in the IPL, CRB3 might not be ubiquitous in this layer. In agreement with this latter notion, CRB3 has already been described in vesicles, such in early endosomes in Caco2 cells, a cell line from epithelial colorectal adenocarcinoma cells [43]. In the retina, the correct transport of synaptic vesicles is needed to ensure correct signal transmission, and some members of the Crumbs complex have been described to be present in presynaptic vesicles, such as MPP4 [26]. Other authors have also described the presence of MPP4 in the plasma membrane of the photoreceptors synaptic terminals, in both cones and rods [27]. In the present work, we show that the colocalization of CRB3 and MPP4 is complete in the OPL. In these photoreceptors presynaptic terminals, CRB3 colocalized with recoverin and it showed partial colocalization with cones markers. Furthermore, the double labeling PNA/CRB3 showed that CRB3 might be present along the base of the cone terminals. Taken together, our findings and previous works, it seems that CRB3 is located in the membrane of presynaptic vesicles, as well as in the plasma membrane of both types of photoreceptors synaptic terminals, where, together with MPP4, it may help in the organization of the presynaptic complex. In addition, the synapses made by both types of photoreceptor cells in the OPL contain VGLUT1, the vesicular transporter of glutamate, which is the major excitatory neurotransmitter in the retina [28], [29] and CRB3. Nevertheless, in the large terminals of photoreceptors and rod bipolar cells, CRB3/VGLUT1 did not show a complete colocalization, which suggests that CRB3 may not be uniformly distributed in synaptic vesicles or may be present in different compartments of the synaptic terminals.

Photoreceptor cells grow during the different stages of embryo development [44], [45], and at P0 both types express recoverin. The recoverin/CRB3 double immunolabeling located the CRB3 protein precisely in the developing synaptic terminals that were organizing the incipient OPL and in the budding IS, where it has been described that recoverin is first expressed [24]. At P4, when only cone terminals are present, since rod terminals invade later [23] we found CRB3 positive processes in the OPL. This developmental data strongly suggest that the cone terminal expresses CRB3, but the PNA or opsin/CRB3 double labeling of the cone IS does not clearly resolve whether this protein is present in the cones IS. Besides, at P4, we found some CRB3 positive cells, but not for recoverin, vitreally to the developing OPL. This suggests that CRB3 could be present in a type of cell other than photoreceptors during the maturation of the retina and/or that CRB3 could be located in photoceptors synaptic terminals before they express recoverin. Taking all these results together and since it has been previously demonstrated the importance of the CRB3 protein in the adhesion properties of several epithelial tissues [7], it is possible that CRB3 could play a role in the establishment of the photoreceptor cells synaptic contacts in the OPL.

We have also analyzed the presence of CRB3 in the processes that organize the IPL. In this layer the processes of the bipolar cells establish synaptic contacts by mean of ribbon synapses, which also express synaptophysin, bassoon and VGLUT1 [29]. The colocalization of CRB3 and synaptophysin labeling would be consistent with CRB3 localization in synaptic vesicles. In contrast, and similarly to what we observed in the OPL, the VGLUT/CRB3 colocalization pattern suggests that CRB3 may not be uniformly distributed in the synaptic vesicles or may be present in different compartments of these terminals. There is also a discrete colocalization of TH and CRB3 in the outermost portion of the IPL, suggesting that CRB3 is present in specific domains, likely synapses, within the processes of the large dopaminergic amacrine cells. In sublamina 5 of the IPL there are processes of the rod bipolar cells that express PKCα [46]. Besides, GNB3 is located in the axon terminals of the cone-ON bipolar cells [47]. Since we found colocalization of CRB3 with VGLUT1, PKCα and GNB3, but not with CR, CB or TH, this therefore suggests that in the IPL, CRB3 is present in rod and in cone bipolar cells. In addition, in some areas of the IPL we found CRB3 labeling to form a ring pattern associated with PKCα rod bipolar cell terminals that could reflect the presence of CRB3 either in the bipolar cell terminal itself or in presynaptic terminals of amacrine cells that provide input to the bipolar cell terminal. Also, most conventional synapses made in the IPL release the inhibitory neurotransmitter GABA [29]. GAD65 and 67 are the two isoforms of the enzyme glutamate decarboxylase, which catalyzes the decarboxylation of glutamate to GABA in the retina [32], [48], [49]. It has been reported that in mammalian retinas, the GABAergic amacrine cells can express either or both GAD isoforms [49], [50]. Using an antibody that recognizes both GAD isoforms, we have shown in the present work that CRB3 colocalizes with most GAD65/67 processes. Hence, taken all this results together, we propose that in the IPL, the CRB3 protein may be broadly and mainly present in GABAergic amacrine cells, as well as in glutamatergic bipolar cells.

### CRB3 and the Crumbs Complex

The members of the CRB family (CRB1, CRB2 and CRB3) organize a protein scaffold involved in the maintenance of adherens junctions in the SAR of the OLM and in the polarization of photoreceptor cells [7], [14], [15]. This protein scaffold is formed by the so-called Crumbs complex, which comprises a group of proteins, including several members of the CRB and the MAGUK protein families.

MPP4, a MAGUK protein, and also a member of the Crumbs complex in the retina, has been detected in the SAR, but its expression is greater in the OPL, in the cones and rod synaptic terminals [25], [26], [27]. In both areas, MPP4 has been located in vesicles [26], as well as in the plasma membrane of the photoreceptors presynaptic terminals [27]. Moreover, in a different study the authors also detected this protein at the level of the photoreceptor connecting cilium and in the IPL in bovine and pig retina [51]. The MPP4 protein is formed by several domains able to mediate protein-protein interactions, such as the L27 or the PDZ domains [51], [52]. Although the role of MPP4 in the mammalian retina is still not clear, a study using Mpp4 knockout mice has shown a loss in the signal of plasma membrane Ca^2+^ ATPases (PMCAs) associated with photoreceptor presynaptic membranes, a perturbation in Ca^2+^ homeostasis and in signal transmission between rods and bipolar cells [27]. The CRB3 protein has a PBM domain in its intracellular portion, which is amenable to binding to the MPP4 PDZ domain.

In summary, although CRB3, as a member of the CRB protein family is known to be involved in the development of adhesion complexes and in the apico-basal determination of several cell types, the distribution and role of this protein in the development of the retina is still poorly understood. In the present work we report evidences that CRB3 is present throughout the inner segment of the photoreceptor cells, where it is especially concentrated in the connecting cilium area in adulthood, as well as during their development. Also, we found this protein in the OPL and in the IPL, during development and in the mature mouse retina, an observation hitherto unreported. The spatial and temporal localization of CRB3 in the mouse retina, together with what is known of the role of CRB3 in the establishment of adhesion complexes and in the ciliogenesis in different types of cells make us suggest that CRB3, apart from being crucial in the maintenance of adhesions in the OLM region, could play a role in the development of the connecting cilium and the plexiform retinal layers. It is possible that at least in the OPL, CRB3 might cooperate with MPP4 in the maintenance of PMCAs, Ca^2+^ homeostasis, and in correct signal transmission. Accordingly, we suspect that CRB3 and MPP4 could be associated to establish the Crumbs complex in both plexiform layers: i.e., in the SAR and in the photoreceptor connecting cilium. However, it would be necessary a more detailed study of the localization of MPP4 in these layers to verify such hypothesis. Also, further studies are needed to demonstrate the functional role of this protein during the development and maintenance of all these elements in the mouse retina.

## Supporting Information

Figure S1 **Peptide competition assay for CRB3 and double immunolabeling CRB3/MPP4 and CRB3/rhodopsin. A–B,** peptide competition assay for CRB3 where all the labeling disappears in retina cryosections at P0 (**A**) and at P4 (**B**). **C–D**, Immunofluorescence (**C**) and peptide competition assay (**D**) for the anti-MPP4 antibody in the adult mouse retina. **C**, MPP4 is present in the photoreceptor synaptic terminals and in the SAR of the OLM (arrowheads and inset). **D**, the MPP4 staining disappears in the peptide competition assay. **E–F**, CRB3 colocalizes with rhodopsin only at the tips of the rods’ IS (arrowheads). OS, photoreceptors’ outer segments; IS, photoreceptors’ inner segments; OLM, outer limiting membrane; ONL, outer nuclear layer; OPL, outer plexiform layer; INL, inner nuclear layer; IPL, inner plexiform layer. Scale bars: 20 µm, 5 µm in inset.
(TIF)

Figure S2 **CRB3 in the Müller glial cells.** Double immunofluorescence for CRB3 (green) and CRALBP (red). **A–C**, CRALBP partially colocalizes with CRB3 in the Müller cells at the level of the OLM (arrowheads). **D–F,** CRALBP and CRB3 do not colocalize in any of the retinal plexiform layers, and the Müller cells processes seem to surround the CRB3 positive profiles (arrows). OLM, outer limiting membrane; OPL, outer plexiform layer; INL, inner nuclear layer; IPL, inner plexiform layer. Scale bars: 20 µm.
(TIF)
