# Correction: Immunocytochemical Evidence of the Localization of the Crumbs Homologue 3 Protein (CRB3) in the Developing and Mature Mouse Retina

**DOI:** 10.1371/annotation/edd43e95-f22f-4c39-9e84-cb246284830c

**Published:** 2013-08-02

**Authors:** Saúl Herranz-Martín, David Jimeno, Antonio E. Paniagua, Almudena Velasco, Juan M. Lara, José Aijón, Concepción Lillo

In Table 1, the catalog number sc-29706 for the antigen CRB3 is incorrect. The correct catalog number is sc-27906. 

