# Supplementary figures and images for: Immunocytochemical Evidence of the Localization of the Crumbs Homologue 3 Protein (CRB3) in the Developing and Mature Mouse Retina
Source: PLoS One. 2012 Nov 30;7(11):e50511. doi: 10.1371/journal.pone.0050511 (PMC3511585; doi:10.1371/journal.pone.0050511)

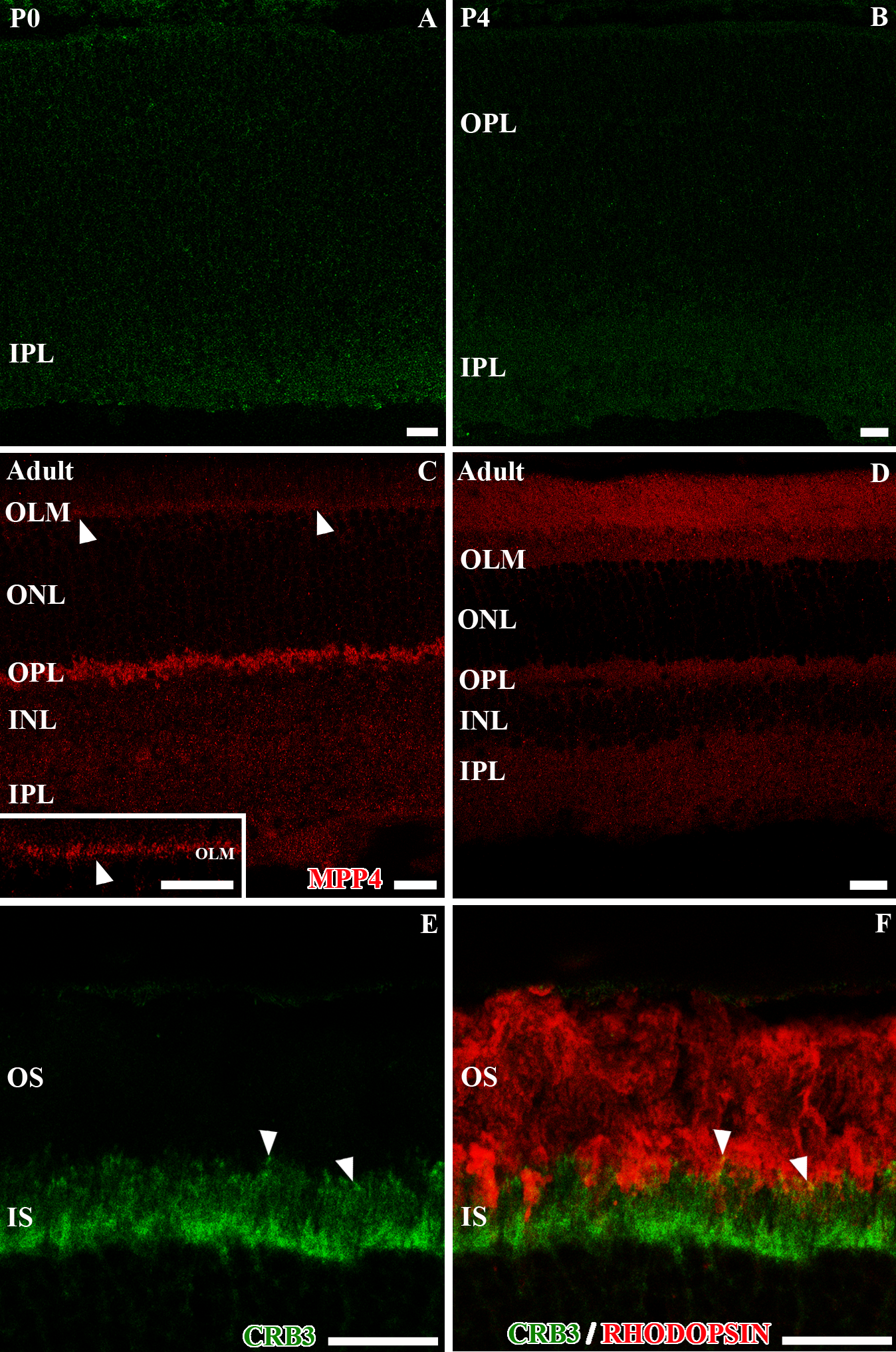

Supplement: Figure S1 — Peptide competition assay for CRB3 and double immunolabeling CRB3/MPP4 and CRB3/rhodopsin. A–B, peptide competition assay for CRB3 where all the labeling disappears in retina cryosections at P0 (A) and at P4 (B). C–D, Immunofluorescence (C) and peptide competition assay (D) for the anti-MPP4 antibody in the adult mouse retina. C, MPP4 is present in the photoreceptor synaptic terminals and in the SAR of the OLM (arrowheads and inset). D, the MPP4 staining disappears in the peptide competition assay. E–F, CRB3 colocalizes with rhodopsin only at the tips of the rods’ IS (arrowheads). OS, photoreceptors’ outer segments; IS, photoreceptors’ inner segments; OLM, outer limiting membrane; ONL, outer nuclear layer; OPL, outer plexiform layer; INL, inner nuclear layer; IPL, inner plexiform layer. Scale bars: 20 µm, 5 µm in inset. (TIF) [file pone.0050511.s001.tif]

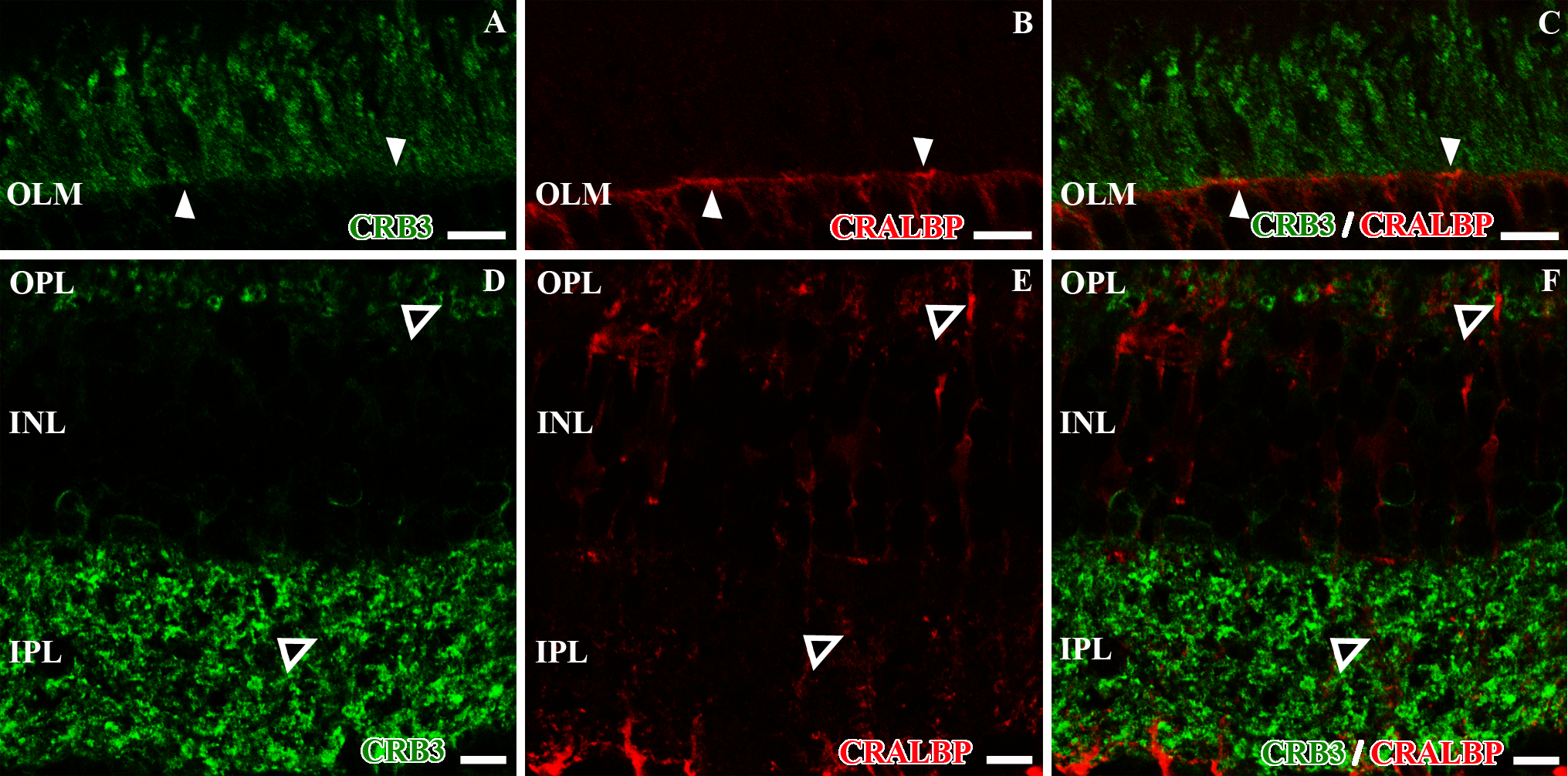

Supplement: Figure S2 — CRB3 in the Müller glial cells. Double immunofluorescence for CRB3 (green) and CRALBP (red). A–C, CRALBP partially colocalizes with CRB3 in the Müller cells at the level of the OLM (arrowheads). D–F, CRALBP and CRB3 do not colocalize in any of the retinal plexiform layers, and the Müller cells processes seem to surround the CRB3 positive profiles (arrows). OLM, outer limiting membrane; OPL, outer plexiform layer; INL, inner nuclear layer; IPL, inner plexiform layer. Scale bars: 20 µm. (TIF) [file pone.0050511.s002.tif]
